# Predicting survival outcomes in advanced pancreatic cancer using machine learning methods

**DOI:** 10.1097/MD.0000000000043904

**Published:** 2025-08-15

**Authors:** İsmet Seven, Cansu Çalişkan, Fahriye Tuğba Köş, Hilal Arslan, Selin Aktürk Esen, Furkan Ceylan, Doğan Uncu

**Affiliations:** a Ankara Bilkent City Hospital, Medical Oncology Clinic, Ankara, Turkey; b Computer Engineering Department, Ankara Yildirim Beyazit University, Ankara, Turkey.

**Keywords:** artificial intelligence, machine learning, pancreatic cancer, predictive models, prognosis, survival

## Abstract

The prognosis for pancreatic cancer (PC) is poor, with a 5-year survival rate of approximately 10%. Methods such as machine learning (ML) can facilitate prognostic assessments by examining complex patterns in patient data that may not be discernible with traditional methods. The aim of this study was to analyze prognostic factors that may influence overall survival in advanced-stage PC using ML methods, and to evaluate the performance of various ML algorithms in predicting patient survival outcomes. A total of 315 patients with inoperable locally advanced or metastatic PC between 2005 and 2023 were included in the study. MATLAB software was used for feature selection. Overall survival was defined as the time from diagnosis to death or last follow-up, and was used as the primary parameter for analysis. The power of 19 clinical and laboratory features of the patients to predict whether patients were deceased, as reflected by importance scores (F-scores), was evaluated using the minimum redundancy–maximum relevance, chi-square, analysis of variance, and Kruskal–Wallis tests as feature selection methods. 24 ML methods were evaluated with these feature selection methods and the results regarding the most effective features were used to predict whether patients were deceased or not. The median age of the patients was 62 years, and 30.5% were women while 69.5% were men. As a result of the analysis of the feature selection methods, the first-line chemotherapy a patient received had the highest F-score in predicting that patient’s survival. Among ML methods, the support vector machine (SVM) kernel method had the highest accuracy rate (87%) in predicting whether patients were deceased. When the feature selection methods were combined with the SVM kernel ML method, patients’ survival statuses could be predicted with an accuracy rate of 87.9%. The SVM kernel method has been demonstrated to show potential as a means of predicting survival for patients with advanced PC. The integration of feature selection with this method yielded high accuracy, thereby underscoring its significance. The findings emphasize the pivotal function of first-line chemotherapy and indicate that ML models have the potential to enhance clinical decision-making and patient care.

## 1. Introduction

Pancreatic cancer (PC) is the 6th leading cause of cancer deaths worldwide.^[1]^ Research suggests that the overall 5-year survival rate for PC is about 10%.^[2]^ It is important to note that survival rates can differ significantly depending on the stage in which the cancer is diagnosed and other varying factors.^[3]^ In cases of advanced PC, the overall survival (OS) rate decreases considerably, with a median survival time ranging from 6 to 11 months.^[4]^ OS rates in advanced PC vary even among patients with the same stage of disease due to variances in tumor biology, response to treatment, and general health.^[5]^ Consequently, while stage is a critical factor in predicting survival, differences in other factors can lead to significant variations in outcomes among patients with similar stages of advanced PC.^[6]^ Accurately identifying and understanding the factors that affect OS in advanced PC is crucial for providing personalized treatment and improving patient outcomes.

Artificial intelligence (AI)-based techniques such as machine learning (ML) are increasingly finding roles in medicine. They are used often for oncological diseases, including applications in diagnosis, treatment selection, and survival prediction. AI has the potential to revolutionize the prediction of OS in advanced PC by taking into account a multitude of unique factors that can influence a patient’s prognosis.^[3]^ By analyzing vast amounts of data, including data on tumor biology, treatment responses, and patient health records, AI algorithms can identify complex patterns and associations that may not be apparent with traditional methods.^[7]^ This advanced level of predictive modeling can lead to more accurate and personalized prognostic assessments for patients, ultimately improving treatment planning and patient outcomes.

Given that over 80% of cases of PC are diagnosed at advanced stages, with nearly half being metastatic, it is projected that this malignancy will become the 3rd leading cause of cancer-related mortality in the European Union in the near future. However, advancement in personalized treatment strategies has been restricted.^[8]^ Current standard-of-care combination chemotherapy has been shown to prolong survival in some patients, yet this is often accompanied by severe adverse effects.^[9,10]^ Accurate prognostic assessment at diagnosis is critical for personalized therapy and for selecting patients for clinical trials. Although the American Joint Committee on cancer staging guidelines are widely used, they provide minimal risk stratification for most people with stage III or IV disease.^[11]^

Other systems, such as the Glasgow prognostic score, which is based on inflammatory and nutritional markers, provide some prognostic value. However, they may not adequately capture the underlying biological complexity of the disease. As evidenced by the following references, numerous nomograms have been developed to predict survival in advanced PC. However, these have often relied on a limited set of patient clinical data.^[12–16]^ It has been demonstrated that the incorporation of multimodal data, encompassing medical imaging, has the capacity to refine survival prediction.^[17,18]^ As ML algorithms become more accessible, they have the potential to detect the complex interactions that influence survival risk.^[19]^

The objective of this study was to analyze prognostic factors that may impact OS in advanced-stage PC using ML methods, and to assess the performance of different ML algorithms in predicting patient survival outcomes.

## 2. Dataset and methods

In this section, we introduce our dataset and summarize methods used in this study. We perform 4 different feature selection methods to identify significant parameters associated with survival. Next, we apply 24 different ML methods (fine tree, medium tree, coarse tree, boosted trees, bagged trees, RUS boosted trees, binary generalized linear model logistic regression, efficient logistic regression, efficient linear support vector machine (SVM), linear SVM, quadratic SVM, cubic SVM, fine Gaussian SVM, medium Gaussian SVM, coarse Gaussian SVM, Gaussian Naive Bayes, kernel Naive Bayes, narrow neural network, medium neural network, wide neural network, bilayered neural network, trilayered neural network, SVM kernel, and logistic regression kernel) were used to predict whether patients were deceased or survivors. In the subsections, we explain these methods.

### 2.1. Dataset

A total of 315 patients with inoperable locally advanced or metastatic PC who were followed in the medical oncology clinics of Ankara Numune Training and Research Hospital and Ankara Bilkent City Hospital between 2005 and 2023 were included in the study. Nineteen separate parameters including the patient’s age at diagnosis, sex, Eastern Cooperative Oncology Group performance status (ECOG PS), complaints at presentation, pancreatic tumor size, location of the tumor in the pancreas, metastasis site(s), number of lines of chemotherapy received, chemotherapy regimen received in the 1st line, number of cycles of chemotherapy received in the 1st line, chemotherapy responses, platelet count, hemoglobin, neutrophil count, albumin, lactate dehydrogenase, carcinoembryonic antigen, carbohydrate antigen 19-9, and lymphocyte count were retrospectively obtained and recorded. Outcome variable shows survival of the patient. The other variables are considered as the input variables. Descriptive analysis of patient characteristics was performed using IBM SPSS Statistics 25.0 (IBM Corp., Armonk). Ethics committee approval was obtained from the Ankara Bilkent City Hospital Medicine and Health Sciences Research Board (decision number: E2-24-6182).

### 2.2. IBM SPSS 25.0

Data were analyzed using IBM SPSS Statistics 25.0 (IBM Corp., Armonk). When the study data were examined in terms of normality assumptions, Kolmogorov–Smirnov values were determined as *P* < .05. Descriptive statistics are presented as frequencies and percentages for categorical variables, and medians (min–max) for continuous variables. Kaplan–Meier method, and log-rank test were employed to analyze survival data. Statistical significance was set at *P* < .05 significance.

### 2.3. Feature selection process

Feature selection has a critical role for identifying survival. In this study, we perform 4 different feature selection methods, the minimum redundancy–maximum relevance (MRMR), chi-square (Chi2), analysis of variance (ANOVA), and Kruskal–Wallis methods to identify significant parameters associated with survival and eliminate redundant or irrelevant variables.

#### 2.3.1. Minimum redundancy–maximum relevance

The MRMR analysis^[20]^ method, one of the methods used for feature selection, was applied in this study. This method aims to select the features that have the highest correlation with the target variable and are minimally related to each other by evaluating the relationships between features in a dataset. MRMR scoring is based on 2 important principles used in the evaluation of each feature: relevance and redundancy. Relevance describes the strong associations of selected features with the target variable, while redundancy reflects the similarity between selected features. The features with the highest MRMR scores are selected for use in the analysis process. This selection aims to reduce the complexity of the model and at the same time preserve important information. MRMR is a feature selection method used to make models more efficient and generalized by reducing the number of features.

#### 2.3.2. Chi2 analysis

The Chi2 analysis^[20]^ method was used in addition to the MRMR method for feature selection. This method was selected with the aim of evaluating the relationships between categorical independent features and categorical dependent features. Chi2 analysis, as a numerical evaluation method, is used to determine the significance of the relationships between features with a *P*-value by testing the deviation of the observed frequencies from the expected frequencies. Chi2 scoring is based on 2 fundamental hypotheses in the analysis process: the null hypothesis and the alternative hypothesis. The null hypothesis (H_0_) holds that there is no relationship between 2 features, while the alternative hypothesis (H_1_) holds that there is a significant relationship between the 2 features.

If the *P*-value is below a certain significance level (usually .05), the null hypothesis is rejected and the existence of a significant relationship between the 2 features is accepted. In this case, the selected features may play an important role in explaining the target feature, and the use of these features aims to make the model more effective and generalized. Chi2 analysis is an effective feature selection method that aims to reduce the number of features and improve the performance of a model by evaluating the significant relationships between features.

#### 2.3.3. Analysis of variance

The ANOVA method^[21]^ was used in this study in addition to the MRMR and Chi2 analysis methods for feature selection. ANOVA, as a statistical method, is a technique used to detect statistically significant differences between group means and thus determine the differences between groups. ANOVA evaluates whether the variance between the means of feature values of different groups is statistically significant compared to the total variance; thus, it determines whether there is a significant difference between the groups according to the obtained *P*-value. If the *P*-value is below a certain significance level (usually .05), it indicates that there is a significant difference between the groups and this difference can be considered in the feature selection process. ANOVA was used in the feature selection process of this study to improve the generalization and optimize the performance of the model.

#### 2.3.4. Kruskal–Wallis method

The Kruskal–Wallis analysis method^[22]^ was used in addition to the MRMR, Chi2, and ANOVA methods for feature selection. This statistical test assesses whether the medians between groups are statistically different. The Kruskal–Wallis test is known as a preferred nonparametric alternative when groups are not normally distributed or do not show equal variance. It determines whether one or more characteristics are statistically significantly different between groups by assessing whether the medians are equal between the groups. A *P*-value below a certain significance level (usually .05) indicates a statistically significant difference between the groups. Therefore, by using the Kruskal–Wallis test in the feature selection process, the differences between groups are evaluated and the features with significant differences can be selected. The inclusion of this method as a nonparametric test in the feature selection process can contribute to increased generalizability of the model and optimized performance.

### 2.4. ML methods

We perform various types of ML methods: decision tree based methods, regression-based method, SVMs, Naïve Bayes, neural networks, and kernels to predict survival. In the following we explain these methods.

The decision tree methods^[23]^ used in ML are modeling techniques for analyzing datasets and extracting patterns. These models consist of decision nodes and branches connecting those nodes. Each node represents a particular feature, and the branches represent the values of the features. A decision tree divides the dataset into subgroups and makes predictions for each subgroup. The learning process takes place by routing instances to the branches of the tree according to their feature values and making decisions at each node. Decision trees are used in classification and regression tasks, and the parameters affect the performance of the model. In ML, such models are effective tools for solving data analysis and classification problems. In this study, 6 decision tree ML modeling methods were used: fine tree, medium tree, coarse tree, boosted trees, bagged trees, and RUS boosted trees.

Regression^[24]^ is a statistical method used in ML to model how a dependent variable is affected by one or more independent variables. The main goal is to understand the relationship between variables and predict the value of the dependent variable. Regression analysis is used to understand patterns in the dataset, predict future values, or understand relationships between variables. Various regression techniques such as linear regression, logistic regression, and decision trees can be selected and applied depending on the requirements of the problem and the characteristics of the dataset. In this study, 2 regression ML modeling methods were used: binary generalized linear model logistic regression and efficient logistic regression.

The SVM approach^[25]^ used in ML is an effective learning algorithm for classification and regression problems. Its main purpose is to classify data points by creating the best hyperplane between 2 classes. In this process, support vectors are important points that maximize marginal gaps between classes. SVM methods can also work successfully in high-dimensional spaces using kernel functions. They exhibit strong performance in classification and regression tasks. In this study, 7 SVM ML modeling methods were used: efficient linear SVM, linear SVM, quadratic SVM, cubic SVM, fine Gaussian SVM, medium Gaussian SVM, and coarse Gaussian SVM.

The naive Bayes approach^[26]^ used in ML is a learning algorithm for classification and probability-based prediction problems. It is based on Bayes’ theorem and simplifies the classification process by assuming independence between features. This algorithm calculates the probabilities of a particular class with combinations of prior knowledge and features, and it is effective in classifying new samples. This method, which is especially preferred in applications such as text classification, stands out with its simple structure and low computational cost. In this study, 2 naive Bayes ML modeling methods, Gaussian Naive Bayes and kernel Naive Bayes, were used.

Neural networks^[27]^ are models used in ML inspired by the biological nervous system. They process input data and produce output using a network of neurons in various layers. Neural networks are used in tasks such as classification, regression, and pattern recognition. In particular, the deep learning subbranch includes multilayer neural networks that are effective in learning complex patterns.

In this study, 5 neural network ML modeling methods were used: narrow neural network, medium neural network, wide neural network, bilayered neural network, and trilayered neural network.

Kernels^[28]^ are mathematical functions used in ML that move datasets into higher-dimensional spaces. They are particularly used with algorithms such as SVM. These functions provide better performance in classification or regression problems by making data that are not linearly separable in low-dimensional spaces become linearly separable in high-dimensional spaces. Using an appropriate kernel allows a model to learn complex structures more effectively, especially for datasets with nonlinear relationships. In this study, 2 kernel ML modeling methods, SVM kernel and logistic regression kernel, were used.

## 3. Results

### 3.1. Experimental setup

All computations were performed on a computer with an 11th Gen Intel® Core™ i7-1185G7 processor running at 3.00 GHz. MATLAB software (MathWorks, Natick) was used for feature selection. The importance scores (F-scores) of the selected features (patient’s age at diagnosis, sex, ECOG PS, complaints at presentation, pancreatic tumor size, location of the tumor in the pancreas, metastasis sites, number of lines of chemotherapy received, chemotherapy regimen received in the 1st line, number of cycles of chemotherapy received in the 1st line, chemotherapy responses, platelet count, hemoglobin, neutrophil count, albumin, lactate dehydrogenase, carcinoembryonic antigen, carbohydrate antigen 19-9, and lymphocyte count) for the prediction of the patients’ survival statuses were obtained using MRMR, Chi2, ANOVA, and Kruskal–Wallis methods.

### 3.2. Data preprocessing

In order to enhance the performance of ML models, the dataset was preprocessed prior to model training. Categorical variables were imputed using the mode, whereas continuous variables were estimated through a regression-based technique, k-nearest neighbor regression.

### 3.3. Performance evaluation

We performed 10-fold cross validation to evaluate the ML methods. In this method, the dataset is randomly divided into 10 equal parts called folds. In each of the 10 iterations, 1-fold is used for the testing, and the remaining 9 folds are used for the training. This process is repeated 10 times, each time with a different fold as the test set. The performance metric (accuracy) from all 10 iterations are averaged to provide an overall estimate of the performance of the ML models. Accuracy of the ML model is calculated as the proportion of correctly classified instances (both survived and not survived) to the total number of predictions. Accuracy is defined as:


$$\begin{array}{l} Accuracy=\frac{TP+TN}{TP+TN+FP+FN} \end{array}$$

where TP (true positives) and TN (true negatives) represent correctly predicted survival and non-survival cases, respectively, while FP (false positives) and FN (false negatives) denote the misclassified instances. This metric provides a straightforward measure of overall classification correctness and was computed for each fold during the 10-fold cross-validation to ensure robust performance assessment.

### 3.4. Experimental results

**Figure 1.**
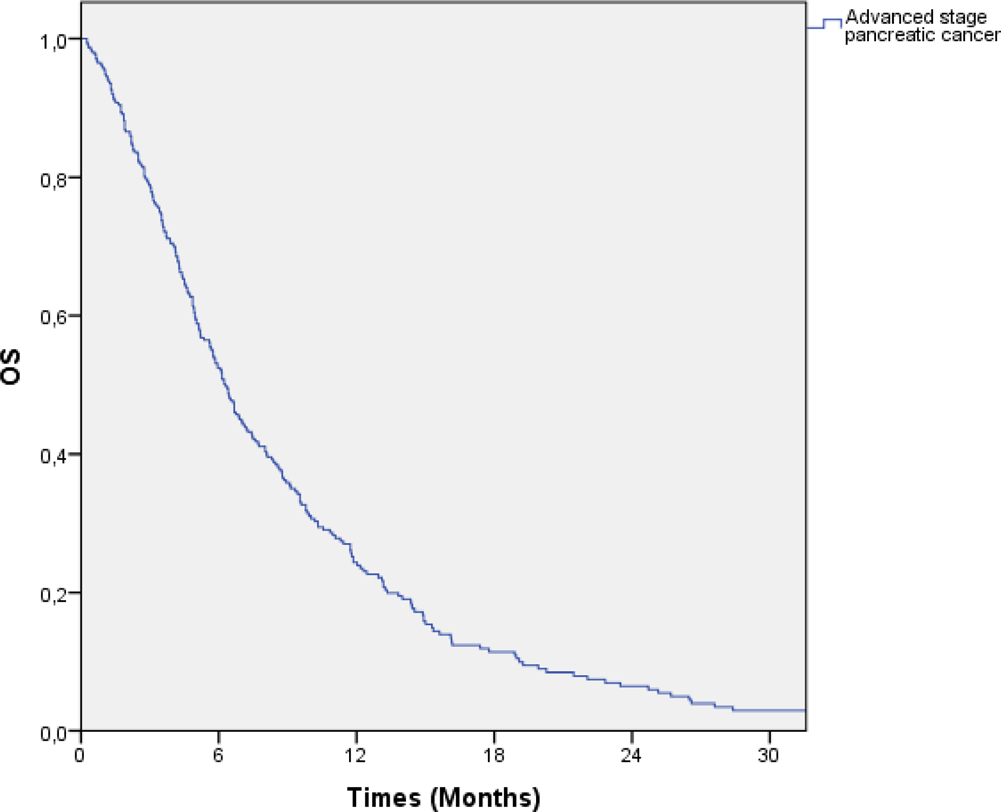
The median overall survival of all patients was 6.2 (5.5–7) months.

A total of 315 patients diagnosed with inoperable locally advanced or metastatic PC were included in this study. 96 (30.5%) of the patients were women and 219 (69.5%) were men, and the median age was 62 (28–89) years. The average primary tumor size was 4 (1–9) cm. 46 (14.6%) patients had an ECOG PS of 0, while 74 (23.6%) had an ECOG PS of 1, 56 (17.8%) had an ECOG PS of 2, 10 (3.2%) had an ECOG PS of 3, and 1 (0.3%) had an ECOG PS of 4. The clinical and laboratory values of the patients are summarized in Table 1. The median OS of all patients was 6.2 (5.5–7) months (Fig. 1).

**Table 1:** Descriptive analysis of clinical characteristics and laboratory values of patients performed with IBM SPSS Statistics 25.0.

		N (%)	Median (minimum–maximum)
Sex	Women	96 (30.5%)	
	Men	219 (69.5%)	
Age (years)			62 (28–89)
Platelets (×10^9^/L)			254 (80–763)
Hemoglobin (g/dL)			12.8 (4.3–16.6)
Lymphocytes (×10^9^/L)			1.50 (0.42–5.66)
Neutrophils (×10^9^/L)			5.35 (1.10–21)
Albumin (g/dL)			3.96 (1.9–5.74)
Lactate dehydrogenase (LDH) (U/L)			242 (34–2098)
Carcinoembryonic antigen (CEA) (ng/mL)			5.24 (0.1–4093)
Carbohydrate antigen 19-9 (CA 19-9) (U/mL)			893 (0.1–700,000)
Eastern Cooperative Oncology Group Performance Score (ECOG PS)	0	46 (24.6%)	
	1	74 (39.6%)	
	2	56 (29.9%)	
	3	10 (5.3%)	
	4	1 (0.5%)	
Complaints	Dyspepsia	8 (5%)	
	Abdominal pain	69 (43.4%)	
	Abdominal pain and jaundice	19 (11.9%)	
	Jaundice	18 (11.3%)	
	Weight loss and jaundice	6 (3.8%)	
	Weight loss	11 (6.9%)	
	Back pain	6 (3.8%)	
	Gastrointestinal bleeding	2 (1.3%)	
	Abdominal pain and weight loss	13 (8.2%)	
	Abdominal pain and back pain	5 (3.1%)	
	Cough-hiccups	1 (0.6%)	
	Chest pain	1 (0.6%)	
Tumor location	Pancreatic head	95 (60.1%)	
	Pancreatic neck	5 (3.2%)	
	Pancreatic body	32 (20.3%)	
	Pancreatic tail	21 (13.3%)	
	Pancreatic body and tail	4 (2.5%)	
	Pancreatic neck-body	1 (0.6%)	
Tumor size (cm)			4 (1–9)
Metastasis location	Non-regional lymph node	21 (7.3%)	
	Liver	46 (16%)	
	Liver and non-regional lymph node	122 (42.5%)	
	Bone and liver	1 (0.3%)	
	Lung and liver	6 (2.1%)	
	Bone and peritoneum	1 (0.3%)	
	Lung	18 (6.3%)	
	Peritoneum, liver, and non-regional lymph node	7 (2.4%)	
	Inoperable, locally advanced	23 (8%)	
	Peritoneum	12 (4.2%)	
	Bone and non-regional lymph node	4 (1.4%)	
	Lung and non-regional lymph node	3 (1%)	
	Peritoneum and non-regional lymph node	3 (1%)	
	Liver, lung, and peritoneum	7 (2.4%)	
	Liver and peritoneum	4 (1.4%)	
	Lung and peritoneum	2 (0.6%)	
	Liver, lung, and non-regional lymph node	3 (1%)	
	Bone, lung, and non-regional lymph node	1 (0.3%)	
	Liver, lung, peritoneum, and non-regional lymph mode	3 (1%)	
How many lines of chemotherapy did the patient receive?	0	27 (17%)	
	1	78 (49.1%)	
	2	35 (22%)	
	3	19 (11.9%)	
First-line chemotherapy	FOLFIRINOX	61 (22.5%)	
	Gemcitabine + nab-paclitaxel	17 (6.3%)	
	Gemcitabine	62 (22.9%)	
	FOLFOX	14 (5.2%)	
	Gemcitabine + cisplatin	115 (42.4%)	
	FUFA	2 (0.7%)	
How many cycles of chemotherapy?			6 (1–7)
Chemotherapy response	Progressive disease	49 (37.4%)	
	Stable disease	59 (45%)	
	Partial response	21 (16%)	
	Complete response	2 (1.5%)	
Overall survival	Alive	42 (13.3%)	
	Deceased	273 (86.7%)	

Analysis was performed for all patients whose data were available in the study.

First, we evaluate the results of the feature selection methods. The F-scores calculated by the MRMR, Chi2, ANOVA, and Kruskal–Wallis methods are given in Table 2. Higher F-score values indicate that the relevant feature is more important and more determinant in a dataset. When Table 2 is analyzed, it is seen that the patients’ first-line chemotherapies have the highest F-scores.

**Table 2:** F-scores reflecting the importance of patient characteristics in predicting patients’ survival statuses using the MRMR, Chi2, ANOVA, and Kruskal–Wallis analysis methods.

MRMR	F-score	Chi2	F-score	ANOVA	F-score	Kruskal–Wallis	F-score
Primary mass size	0	Hemoglobin	0.0776	Metastasis location	0.1307	ECOG PS	0.0869
Hemoglobin	0	Age	0.575	ECOG PS	0.3071	CA 19-9	0.0973
Sex	0	Sex	0.848	Hemoglobin	0.3126	Age	0.2168
Platelets	0	Neutrophils	1.0222	CA 19-9	0.3554	Platelets	0.5532
CA 19-9	0	CA 19-9	1.3251	Age	0.371	Sex	0.8463
Albumin	0	Primary mass size	1.3442	CEA	0.6396	Hemoglobin	0.9668
Sex	0.0001	Platelets	1.6856	Sex	0.8443	Chemotherapy response	1.8224
Tumor location	0.0002	CEA	1.965	Platelets	1.3294	Lymphocytes	1.8225
Number of lines of chemotherapy	0.0002	Albumin	2.1325	Albumin	1.482	Primary mass size	1.8582
Complaints	0.0002	Lymphocytes	2.9531	Primary mass size	1.7314	Albumin	2.1266
Number of cycles of chemotherapy	0.0002	Tumor location	2.9679	Tumor location	1.7777	Metastasis location	2.753
Neutrophils	0.0003	Complaints	3.3888	Lymphocytes	1.855	Neutrophils	2.9619
Chemotherapy response	0.0003	ECOG PS	4.4194	Chemotherapy response	2.5243	Tumor location	3.1575
Metastasis location	0.0003	LDH	4.7101	Neutrophils	2.7218	CEA	4.656
CEA	0.0003	Number of lines of chemotherapy	7.2615	Complaints	3.3574	Complaints	5.16
ECOG PS	0.0005	Number of cycles of chemotherapy	8.1784	LDH	5.7368	Number of lines of chemotherapy	7.2988
LDH	0.0029	Metastasis location	8.3732	Number of lines of chemotherapy	6.1043	LDH	8.0695
Lymphocytes	0.0037	Chemotherapy response	9.8239	Number of cycles of chemotherapy	10.7527	Number of cycles of chemotherapy	8.6998
First-line chemotherapy	0.0776	First-line chemotherapy	22.8596	First-line chemotherapy	23.0372	First-line chemotherapy	20.1004

ANOVA = analysis of variance, CA 19-9 = carbohydrate antigen 19-9, CEA = carcinoembryonic antigen, Chi2 = chi-square, ECOG PS = Eastern Cooperative Oncology Group performance status, LDH = lactate dehydrogenase, MRMR = minimum redundancy–maximum relevance.

In addition, 24 ML methods were applied. In Table 3, the accuracy values of the 24 ML methods without feature selection are provided. The ML method with the highest accuracy value was found to be SVM Kernel.

**Table 3:** Accuracy rates of machine learning methods in predicting survival status of patients using all data without feature selection.

Fine tree	81
Medium tree	81
Coarse tree	81.9
Binary GLM logistic regression	81
Efficient logistic regression	86.7
Efficient linear SVM	86.7
Gaussian Naive Bayes	77.8
Kernel Naive Bayes	80.3
Linear SVM	86.3
Quadratic SVM	85.4
Cubic SVM	86.7
Fine Gaussian SVM	86.7
Medium Gaussian SVM	86.7
Coarse Gaussian SVM	86.7
Boosted trees	84.4
Bagged trees	85.4
RUS boosted trees	77.5
Narrow neural network	84.1
Medium neural network	84.8
Wide neural network	85.7
Bilayered neural network	82.2
Trilayered neural network	82.5
SVM kernel	**87**
Logistic regression kernel	86.7

GLM = generalized linear model, SVM = support vector machine.

The 24 ML methods were also evaluated by the MRMR, Chi2, ANOVA, and Kruskal–Wallis analysis methods. The highest accuracy value was obtained for the SVM Kernel ML method using the MRMR, Chi2, and ANOVA analysis methods (Table 4).

**Table 4:** Accuracy rates in predicting survival status as a result of evaluations of the SVM Kernel machine learning method with the MRMR, Chi2, ANOVA, and Kruskal–Wallis analysis methods.

Machine learning method	Analysis method	Feature count	Features	Accuracy
SVM kernel	MRMR	9	Number of cycles of chemotherapy Neutrophils Chemotherapy response Metastasis location CEA ECOG PS LDH Lymphocytes First-line chemotherapy	87.9%
SVM kernel	Chi2	10	Lymphocytes Tumor location Complaints ECOG PS LDH Number of lines of chemotherapy Number of cycles of chemotherapy Metastasis location Chemotherapy response First-line chemotherapy	87.9%
SVM kernel	ANOVA	3	Number of lines of chemotherapy Number of cycles of chemotherapy First-line chemotherapy	87.9%
SVM kernel	Kruskal–Wallis	8	Neutrophils Tumor location CEA Complaints Number of lines of chemotherapy LDH Number of cycles of chemotherapy First-line chemotherapy	87.3%

ANOVA = analysis of variance, CEA = carcinoembryonic antigen, Chi2 = chi-square, ECOG PS = Eastern Cooperative Oncology Group performance status, LDH = lactate dehydrogenase, MRMR = minimum redundancy–maximum relevance, SVM = support vector machine.

## 4. Discussion

Since the OS rate in PC is quite low, it is of great importance to predict the survival of patients with this cancer.^[2]^ While many studies have aimed to shed light on tests such as tumor biology and genomic analysis to predict survival, the use of AI-based ML methods in this field is now increasing.^[29]^ In our study, with all 4 applied analysis methods (MRMR, Chi2, ANOVA, and Kruskal–Wallis methods) used to identify patient characteristics that are effective in predicting the survival status of patients with advanced-stage PC, the first-line chemotherapy that patients received had the highest F-scores. Additionally, among the considered AI ML methods, the SVM Kernel method had the highest accuracy rate (87%) in predicting whether patients were deceased or survivors. When feature selection was performed with patient data using the MRMR, Chi2, ANOVA, and Kruskal–Wallis analysis methods in combination with the SVM Kernel ML method, which had the highest accuracy rate, it was possible to predict the survival status of patients with accuracy rates of 87.9%, 87.9%, 87.9%, and 87.3%, respectively. To our knowledge, this is the 1st study to evaluate prediction of the survival of patients with advanced PC using these methods. However, a previous study demonstrated the potential of AI-derived morphological signatures, such as V-FFX and V-GNP, being strongly associated with treatment outcomes for first-line FOLFIRINOX (FFX) and gemcitabine + nab-paclitaxel (GNP) regimens, respectively.^[30]^

A study by Baek et al^[1]^ investigated the application of neural network models, and more specifically deep learning architectures, in analyzing complex patterns and predicting survival probabilities in cases of advanced PC. Their study demonstrated the robust predictive capabilities of multilayer neural networks in capturing nuanced relationships between treatment variables and patient outcomes. In the Cancer Genome Atlas, survival and recurrence rates were found to be significantly different in pancreatic adenocarcinoma patients with mutations in the *DKN2A*, *TP53*, *TTN*, *KCNJ18*, and *KRAS* genes, which are mutated in the early stages of tumor formation and have a high cellular prevalence, compared to patients without mutations in those candidate genes. Additionally, the integration of AI, hyperpolarized metabolic-magnetic resonance imaging, and multimodal imaging information has the potential to develop real-time biomarkers for early detection, the assessment of aggressiveness, and evaluation of the early efficacy of therapy in pancreatic ductal adenocarcinoma.^[31]^

Among the 24 different ML techniques employed for prediction in advanced PC, SVM kernel demonstrated the highest level of accuracy. This finding suggests that SVM kernel could be a promising approach for accurately predicting factors affecting OS in advanced PC. Similar studies have indicated that the SVM kernel ML method, when integrated with specific clinical and laboratory features, has a high level of accuracy in predicting factors influencing OS across multiple types of cancer, including breast cancer, lung cancer, and liver cancer.^[32–34]^ An improved SVM model based on the cuckoo search algorithm predicts recurrence location by evaluating 7 characteristics including basic indicators, immune indicators, tumor indicators, nutritional indicators, psychological indicators, microenvironment indicators, aerobic exercise, and advanced work for 776 patients diagnosed with hepatocellular cancer.^[32]^ Recurrence location was predicted correctly at a rate of 95.7% in that study. In another study, a model to predict whether a breast lump is benign or malignant was created using the SVM ML technique, and it was determined that SVM outperformed methods such as logistic regression, decision trees, and random forests with a 96% accuracy rate in its predictions.^[33]^ In a different study, a model predicting survival in lung cancer was established with features such as tumor grade, tumor size, sex, age, stage, and number of primary cancers and analyzed with methods such as decision trees, gradient boosting machines, and SVM.^[34]^ Gradient boosting machines was found to be the most accurate among the models, and while the SVM model performed poorly, statistical analysis showed that SVM was the only model that produced a distinctive output.^[34]^

Sun et al^[35]^ found that the SVM algorithm based on parameter optimization had a high accuracy rate in predicting survival risks for patients based on 9 blood indexes for esophageal cancer. Their model achieved accuracy above 90%. Another study used 5 different ML algorithms to perform survival predictions in colorectal cancer. Each model provided predictions with approximately 77% accuracy and an area under the curve value close to 0.86 according to the stage of the disease.^[36]^ Tong et al^[6]^ used an artificial neural network to predict the 8-month survival rates of patients with advanced PC according to clinical and biochemical features. Their artificial neural network model with the best results achieved an area under the curve value of 0.92. Walsack et al^[37]^ used records from 283 patients to train and validate several artificial neural network models, aiming for a high sensitivity rate to predict 7-month survival. The selected model achieved approximately 90% sensitivity.

The strength of the article is that it demonstrates the potential of advanced ML, in particular the SVM Kernel method, to accurately predict survival in patients with advanced PC. The study shows that feature selection combined with the SVM Kernel approach achieves high accuracy rates, highlighting the value of this approach for reliable outcome prediction. The results also highlight the critical role of first-line chemotherapy in determining survival and suggest that the use of ML models with relevant clinical data can serve as powerful tools to support clinical decision making and patient care.

This study had some limitations. The fact that it was retrospective was the biggest limitation. Due to the demographic framework of our study population, it may not capture global diversity. Therefore, extrapolating our results to a global scale would require rigorous validation across heterogeneous demographic groups. However, it is also important to note that this is one of the rare studies to date on the prediction of survival using ML methods.

## 5. Conclusion

The study demonstrates the promise of ML, particularly the SVM kernel method, in accurately predicting survival for patients with advanced PC. Combining feature selection with the SVM kernel approach achieved high accuracy, highlighting the value of this approach for reliable outcome prediction. The findings highlight the critical role of first-line chemotherapy in determining survival, and suggest that the use of ML models with relevant clinical data can serve as powerful tools to support clinical decision making and patient care.

## Author contributions

**Data curation:** İsmet Seven, Furkan Ceylan.

**Formal analysis:** Cansu Çalişkan.

**Investigation:** Selin Aktürk Esen.

**Methodology:** Hilal Arslan.

**Project administration:** Fahriye Tuğba Köş.

**Resources:** Selin Aktürk Esen.

**Supervision:** Doğan Uncu.
